# Structure Evolution and Bonding Inhomogeneity toward High Thermoelectric Performance in Cu_2_CoSnS_4–*x*_Se_*x*_ Materials

**DOI:** 10.1021/acs.chemmater.3c00586

**Published:** 2023-06-07

**Authors:** Taras Parashchuk, Oleksandr Cherniushok, Oleksandr Smitiukh, Oleg Marchuk, Krzysztof T. Wojciechowski

**Affiliations:** † Thermoelectric Research Laboratory, Department of Inorganic Chemistry, Faculty of Materials Science and Ceramics, AGH University of Science and Technology, Mickiewicza Ave. 30, 30-059 Krakow, Poland; ‡ Department of Chemistry and Technology, Volyn National University, Voli Ave 13, Lutsk 43025, Ukraine

## Abstract

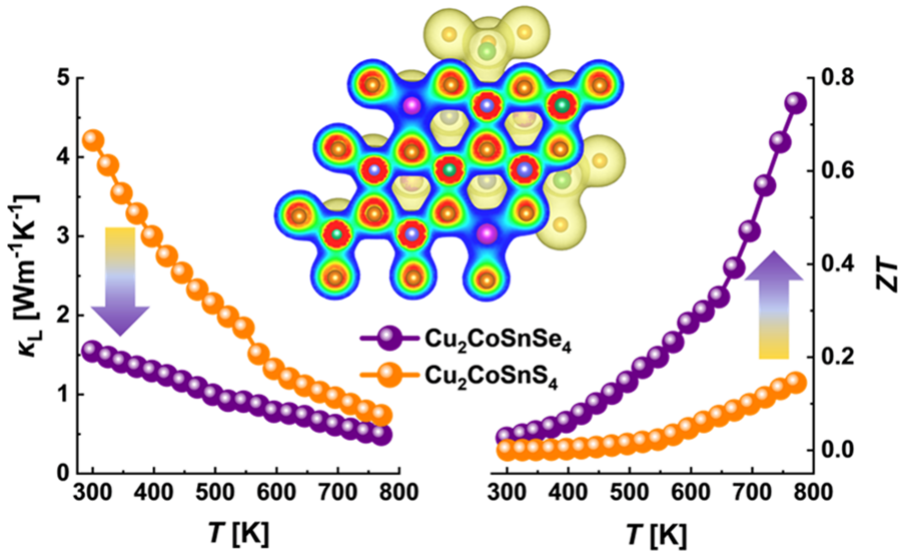

Lightweight diamond-like structure (DLS) materials are excellent candidates for thermoelectric (TE) applications due to their low costs, eco-friendly nature, and property stability. The main obstacles restricting the energy-conversion performance by the lightweight DLS materials are high lattice thermal conductivity and relatively low carrier mobility. By investigating the anion substitution effect on the structural, microstructural, electronic, and thermal properties of Cu_2_CoSnS_4–*x*_Se_*x*_, we show that the simultaneous enhancement of the crystal symmetry and bonding inhomogeneity engineering are effective approaches to enhance the TE performance in lightweight DLS materials. Particularly, the increase of *x* in Cu_2_CoSnS_4–*x*_Se_*x*_ makes the DLS structure with the ideal tetrahedral bond angles of 109.5° favorable, leading to better crystal symmetry and higher carrier mobility in samples with higher selenium content. In turn, the phonon transport in the investigated DLS materials is strongly disturbed due to the bonding inhomogeneity between anions and three sorts of cations inducing large lattice anharmonicity. The increase of Se content in Cu_2_CoSnS_4–*x*_Se_*x*_ only intensified this effect resulting in a lower lattice component of the thermal conductivity (κ_L_) for Se-rich samples. As a result of the enhanced power factor *S*^2^ρ^–1^ and the low κ_L_, the dimensionless thermoelectric figure of merit *ZT* achieves a high value of 0.75 for Cu_2_CoSnSe_4_ DLS material. This work demonstrates that crystal symmetry and bonding inhomogeneity play an important role in the transport properties of DLS materials and provide a path for the development of new perspective materials for TE energy conversion.

## 1 Introduction

Demand for renewable energy is growing continuously due to the reduction in natural resources and problems with environmental pollution^1,2^ provoking extensive investigations of the new technologies for alternative renewable energy.^3^ Because of the unique possibility to interconvert thermal energy and electricity, particular attention of the research community is dedicated to thermoelectric technologies that can be used for the production of electrical energy^4^ and refrigeration.^5^ For over half a century, thermoelectric conversion has been successfully used for powering NASA missions.^6^ However, the high materials costs and toxicity of constituent elements make the best thermoelectrics (e.g., Bi_2_Te_3_, PbTe, GeTe, SiGe) too expensive for broad commercial use.^7−10^ Therefore, the search for low-cost and environmentally friendly materials is among the main tasks of thermoelectric (TE) materials engineering and TE energy development.

The ability of TE materials to convert energy is determined by the dimensionless thermoelectric figure of merit, *ZT* = *S*^2^*T*/ρ(κ_L_ + κ_e_), where *S* is the Seebeck coefficient, ρ is the electrical resistivity, *T* is the absolute temperature, and κ_L_ and κ_e_ are the lattice and electronic components of the thermal conductivity κ, respectively.^11,12^ Consequently, the highly efficient energy conversion by TE materials can be obtained only in case of high power factor (*S*^2^/ρ) and low total thermal conductivity κ (κ = κ_L_ + κ_e_).^12^ However, this is not a simple task, as the Seebeck coefficient *S*, electrical resistivity ρ, and electronic thermal conductivity κ_e_ are interconnected through the carrier concentration *n*_H_ and features of the band structure.^13^ Particularly, the decrease of electrical resistivity ρ of the material is usually accompanied by the simultaneous decrease of the Seebeck coefficient *S* and the rise of the electronic thermal conductivity κ_e_ leading to lower *ZT*. Therefore, improvement of the TE figure of merit *ZT* is still one of the main challenges for modern thermoelectric science.

In the past decades, the large efforts of the thermoelectric community are devoted to the increase of the power factor through the optimization of carrier concentrations,^14^ band engineering,^15^ resonance scattering,^16^ and advanced electronic structure engineering.^17,18^ The successful reduction of the lattice thermal conductivity was attained using grain boundaries engineering,^19,20^ nanostructuring,^21,22^ point defects engineering,^23,24^ lattice anharmonicity,^25,26^ and lattice softening.^27−29^

**Figure 1:**
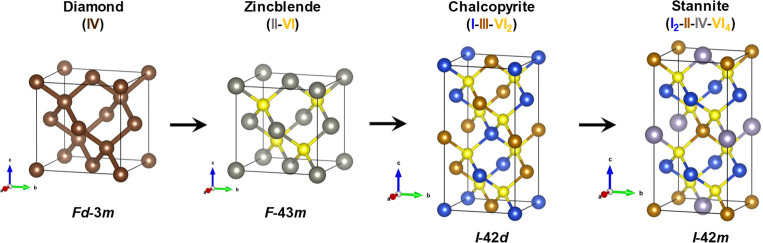
Zincblende, chalcopyrite, and stannite structures as the derivatives of the diamond lattice. All structures are tetrahedrally coordinated with a one-to-one cation-to-anion ratio (e.g., ZnS, CuFeS_2_, Cu_2_CoSnS_4_).

The performed screening, which considers a wide spectrum of requirements for highly efficient thermoelectric materials,^12^ brings our attention to the large family of compounds with diamond-like structure (DLS). The DLS semiconductors are a chemically rich family of compounds that can be derived from the cubic diamond/silicon structure type through simple electron counting rules.^30,31^ From the diamond structure type as a starting point, the binary, ternary, and quaternary DLS materials can be composed (Figure 1). In the diamond-like compounds, only half of the tetrahedral structural voids are filled with cations and all octahedral voids are unoccupied.^32^ The binary DLS materials crystallize in the cubic zinc blende (sphalerite) or hexagonal wurtzite structures.^33,34^ Instead, the ternary and quaternary DLS materials usually have tetragonal structures.^35^

One of the most established and well-investigated DLS thermoelectric materials is silicon–germanium alloys.^36^ Due to favorable electronic transport, good mechanical properties, and high thermal stability, these alloys have been successfully used to produce high-temperature thermoelectric modules for space applications.^37^ In turn, as a result of the high thermal conductivity and relatively large bandgap (*E*_g_ ≈ 0.7–1.2 eV^38^) at 298 K, Si_*x*_Ge_1–*x*_-based alloys show rather poor thermoelectric performance at medium temperatures. The other disadvantage of these alloys is the high price of germanium which also limits their wide utilization in industrial applications.

While the binary DLS materials are widely discussed in literature,^39^ the transport properties of the ternary and quaternary compounds are only starting to be investigated.^35^ Existing literature on some selenide materials (e.g., Cu_2_FeSnSe_4_, Cu_2_CdSnSe_4_, Cu_2_ZnGeSe_4_) indicates that the quaternary materials possess significantly reduced lattice thermal conductivity (∼2 W m^–1^ K^–1^ at 300 K)^40−42^ as compared to the binary and ternary compounds (∼10 W m^–1^ K^–1^ at 300 K).^42^ At the first site, the reduction of the lattice thermal conductivity is consistent with the more complex crystal cells and the increased potential for disorder. Nevertheless, such an explanation can tell nothing about the large difference in the κ_L_ values for different DLS materials with the same number of constituent elements.

Among the DLS materials, due to low-cost and environmentally friendly chemical composition, our attention was drowned on Cu_2_CoSnQ_4_ (Q = S, Se) quaternary compounds. Both of them have a zinc-blende-derived stannite structure and belong to the tetragonal system with the space group *I*4̅2*m*.^43^ The thermoelectric properties of Cu_2_CoSnSe_4_ were reported by Song et al.,^44^ where the high *ZT* = 0.7 at 850 K for this compound was explained by the high band degeneracy and complex chemical bonding between anions and cations. Zhang et. al reported *ZT* around 0.2 at 800 K for pristine Cu_2_CoSnS_4_ and around 0.8 at 800 K for Cu_2.15_Co_0.8_Mn_0.05_SnS_4_ with the optimized carrier concentration.^45^ The authors suggested the weakening of covalent bonding and the appearance of the CuCo_2_S_4_ highly conductive metal-like second phase to be the main reasons for the enhanced *ZT*. However, such clarification, which is based on the interplay between several effects, is not conducive to explaining the relationship between the crystal structure of DLS materials and their thermoelectric properties.

In this work, we investigated the effect of S/Se substitution on the crystal structure and thermoelectric properties of Cu_2_CoSnS_4–*x*_Se_*x*_ alloys. The performed analysis suggests that the higher crystal symmetry of selenide compared to sulfide promotes higher carrier mobility and significantly facilitates electronic transport. In turn, the bonding inhomogeneity and lattice anharmonicity are mainly responsible for the low thermal conductivity in the investigated alloys. As a result, a high *ZT* of 0.75 was achieved for the low-cost and eco-friendly Cu_2_CoSnSe_4_ compound, opening the potential of this material for thermoelectric applications. Moreover, the performed investigation of the crystal structure and phase equilibria combined with the electronic and thermal transport properties gives the wide spectra of knowledge necessary for improving the energy conversion performance in the whole DLS material family.

## 2 Experimental Details

### 2.1 Materials and Synthesis

Samples with the nominal compositions of Cu_2_CoSnS_4–*x*_Se_*x*_ (*x* = 0; 1; 2; 3; 4) were prepared by melting high-purity Cu (shot, 99.99%), Co (shot, 99.99%), Sn (shot, 99.999%), S (shot, 99.999%), and Se (shot, 99.999%) in quartz containers evacuated to a residual pressure of 10^–5^ mbar. The total mass of each sample was 3 g. The ampules with the stoichiometric mixtures of elements were heated to 1423 K at the rate of 12 K/h, kept at this temperature for 4 h, and cooled to room temperature at the same rate. To improve the homogeneity of the synthesized materials, the obtained ingots were crushed into fine powders, compacted using a cold press, heated in evacuated quartz ampules up to 773 K with the rate of 12 K/h, annealed at this temperature for 500 h, and quenched in cold water without breaking the containers.

### 2.2 Sintering

After the annealing process, the samples were crushed into fine powders by hand milling in an agate mortar and then densified by the Spark Plasma Sintering (SPS) technique at 823–873 K depending on sample composition for 60 min in 12.8 mm diameter graphite dies under an axial compressive stress of 45 MPa in an argon atmosphere. The heating and cooling rates were 70 and 20 K/min, respectively. It is expected that the relatively long-term sintering time should also positively affect the homogeneity of the investigated samples. The compacted pellets with a diameter of 12.8 mm and height of ∼2 mm were obtained and polished for transport properties measurements. The density of all pellets was higher than 97% of the crystallographic density.

### 2.3 Powder X-ray Diffraction, Thermal Analysis, and Scanning Electron Microscopy (SEM)

Phase identification was performed with a BRUKER D8 Advance X-ray diffractometer using Cu Kα radiation (λ = 1.5418 Å, Δ2θ = 0.005°, 2θ range of 10–120°) with Bragg–Brentano geometry. Rietveld refinement of the crystal structure was carried out using the WinCSD program package.^46^

Thermal analysis of the investigated materials was performed on Differential Scanning Calorimetry equipment (Netzsch DSC 404 F3 Pegasus) using a sample mass of ∼10 mg in Al crucibles covered by a lid with a heating rate of 10 K/min under a helium flow.

For SEM and Energy-Dispersive X-ray Spectroscopy (EDS) analyses, samples were embedded in conductive resin and subsequently polished, finally using 0.1 μm diamond powder in a slurry. The analysis of the chemical composition was performed using a Scanning Electron Microscope (JEOL JSM-6460LV Scanning Electron Microscope) equipped with EDS. The distribution of the Seebeck coefficient on the sample’s surface was analyzed using the Scanning Thermoelectric Microscope with a resolution of 1 μm.

### 2.4 Electrical and Thermal Transport Properties

The Seebeck coefficient *S* and electrical resistivity ρ were measured using commercial apparatus NETZSCH SBA 458 Nemesis. Measurements were spent in argon flow over the temperature range of 298–773 K. Thermal diffusivity α_D_ was measured on NETZSCH LFA 457 equipment, and the specific heat capacity *C*_p_ was estimated with the help of the Dulong–Petit limit. Before measurements, all samples were first spray-coated with a thin layer of graphite to minimize errors from the emissivity of the material and laser beam reflection caused by a shiny pellet surface. Thermal conductivity was calculated using the equation κ = *dC*_p_α_D_, where *d* is the density obtained by the Archimedes principle at the discs from SPS. The uncertainty of the Seebeck coefficient and electrical resistivity measurements was 7% and 5% respectively, whereas the uncertainty of thermal diffusivity measurements was 3%. The combined uncertainty for the determination of the thermoelectric figure of merit *ZT* is assumed to be equal to 20%.^47^ The Hall effect was investigated by applying the four-probe method in constant electric and magnetic fields (*H* = 0.9 T) and current through a sample of 50 mA. The uncertainty of Hall measurements was ∼10%. The speed of sound was measured at room temperature using the ultrasonic flaw detector Olympus Epoch 650.

### 2.5 Computational Details

Quantum chemical (QC) calculations were performed using the Firefly QC program package,^48^ which is based on the GAMESS (US) source code.^49^ The calculations were performed based on the hybrid functional B3LYP that used the Becke GGA functional for the exchange energy and the Lee–Yang–Parr GGA functional for the correlation energy. For the calculations, we employed lattice parameters, symmetry information, and atomic coordinates obtained during the crystal structure refinement of the Cu_2_CoSnS_4_ and Cu_2_CoSnSe_4_ compounds. The basis sets for the self-consistent calculations can be obtained from the authors. The analysis of the chemical bonding for the investigated materials was performed by the meaning of the electron localization function (ELF).^50^ To perform the necessary topological analysis of the electron density and ELF, we used ChemCraft^51^ and VESTA^52^ software.

## 3 Results and Discussion

### 3.1 Crystal Structure and Bonding Analysis

**Figure 2:**
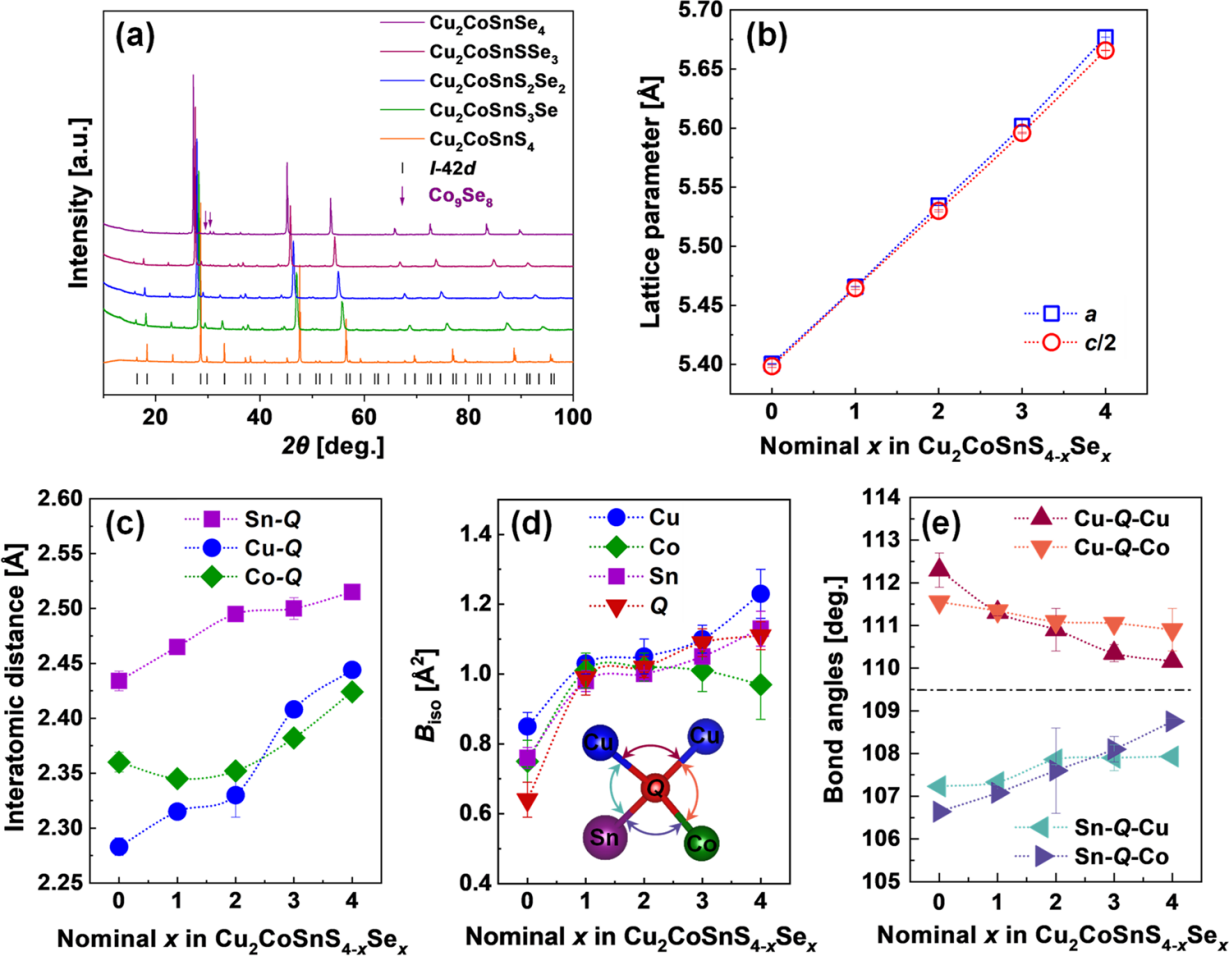
(a) Powder X-ray diffraction patterns, (b) lattice parameters, (c) interatomic distances, (d) atomic displacement parameters *B*_iso_, and (e) metal–chalcogen (Q)–metal bond angles for Cu_2_CoSnS_4–*x*_Se_*x*_ alloys.

Structural analysis of synthesized Cu_2_CoSnS_4–*x*_Se_*x*_ samples was performed using powder X-ray diffraction (Figure 2a). The shift of reflections’ positions toward lower 2θ values with increasing *x* content in Cu_2_CoSnS_4–*x*_Se_*x*_ samples indicates lattice expansion due to the higher ionic radius of Se^2–^ (1.93 Å) in comparison to S^2–^ (1.82 Å).^53^ The crystal structure of the synthesized alloys is described by tetragonal symmetry. After making a phase analysis, we established that the phases do not contain admixtures except for Co_9_Se_8_. The lattice parameters of Cu_2_CoSnS_4–*x*_Se_*x*_ samples after annealing were accurately determined using the WinCSD program package,^46^ and the results are shown in Figure 2b. The trends of the lattice parameter vs chemical composition show an isotropic increase of the *a* and *c* values with the increase of selenium content following Vegard’s law (Figure 2b). The strict trend in lattice parameters and constant *c*/*a* ratio corroborate a homogeneous chalcogen distribution within each sample and the successful substitution of sulfur by selenium in Cu_2_CoSnS_4–*x*_Se_*x*_.

The interatomic distances (Sn–Q, Cu–Q, and Co–Q (Q = S, Se)) for the investigated Cu_2_CoSn_4–*x*_Se_*x*_ solid solutions are increasing with the rise of selenium content *x* (Figure 2c), which can be explained by the larger ionic radius of Se^2–^ (1.93 Å) compared to the ionic radius of S^2–^ (1.82 Å).^53^ In turn, the different slopes of Sn–Q, Cu–Q, and Co–Q interatomic distances, as well as the growth of ADPs in the S → Se series, may also be an indicator of the weakening of bonds. Particularly, the ADP of Q-anions increases in the S → Se series (Figure 2d) contradicting the statement that the heavier atoms vibrate with a lower amplitude. The sharpest increase of the *B*_iso_ parameter can be observed for Cu and Q atoms, highlighting the largest bond softening between these atoms in the structure. We also should mention that the Cu–Q–Cu and Cu–Q–Co angles decrease, while Sn–Q–Cu and Sn–Q–Co angles increase with the rise of *x* in Cu_2_CoSn_4–*x*_Se_*x*_ solid solutions. It is even more interesting that all angles in the structure are approaching the ideal tetrahedral value of 109.5° in the S → Se direction, which is an indicator of higher symmetry of the structure (Figure 2e). As a result, one can expect an increase in carrier mobility with the rise of the selenium content in Cu_2_CoSn_4–*x*_Se_*x*_ solid solutions.^40,44^

Table 1 shows the crystallographic information and physical parameters obtained using the Rietveld refinement of the powder XRD patterns (Figure S1). For the refinement of the crystal structure, we used the model with tetragonal symmetry (space group *I*4̅2*m*). All reflections were successfully indexed in the space group *I*4̅2*m*. As can be seen in Table 1, the lattice parameters, cell volumes, and crystallographic densities increase with the rise of selenium content due to the higher ionic radius of Se^2–^ (1.93 Å) compared with the ionic radius of S^2–^ (1.82 Å).^53^

**Table 1:** Crystallographic Information of Cu_2_CoSnS_4–*x*_Se_*x*_ Solid Solutions

Nominal composition	Cu_2_CoSnS_4_	Cu_2_CoSnS_3_Se	Cu_2_CoSnS_2_Se_2_	Cu_2_CoSnSSe_3_	Cu_2_CoSnSe_4_
Formula weight	432.995	479.901	526.807	573.713	620.619
Space group	*I*4̅2*m*				
*a* (Å)	5.4002(1)	5.4657(3)	5.5345(2)	5.6018(1)	5.6769(1)
*c* (Å)	10.7960(3)	10.929(1)	11.0600(8)	11.1919(5)	11.3315(2)
*V* (Å^3^)	314.84(2)	326.48(7)	338.78(5)	351.21(3)	365.19(2)
Calculated density(g cm^–3^)	4.57	4.88	5.16	5.42	5.64
Absorption coefficient, cm^–1^	706.56	725.55	741.43	756.91	767.72
Data range 2θ (deg)	10–100				
Refinement mode	Full profile				
*R*_*i*_	0.063	0.030	0.024	0.038	0.036
*R*_*p*_	0.107	0.131	0.102	0.091	0.078
*R*_*wp*_	0.025	0.027	0.028	0.022	0.036
Goodness of fit	1.96	2.64	1.63	1.49	1.91

**Figure 3:**
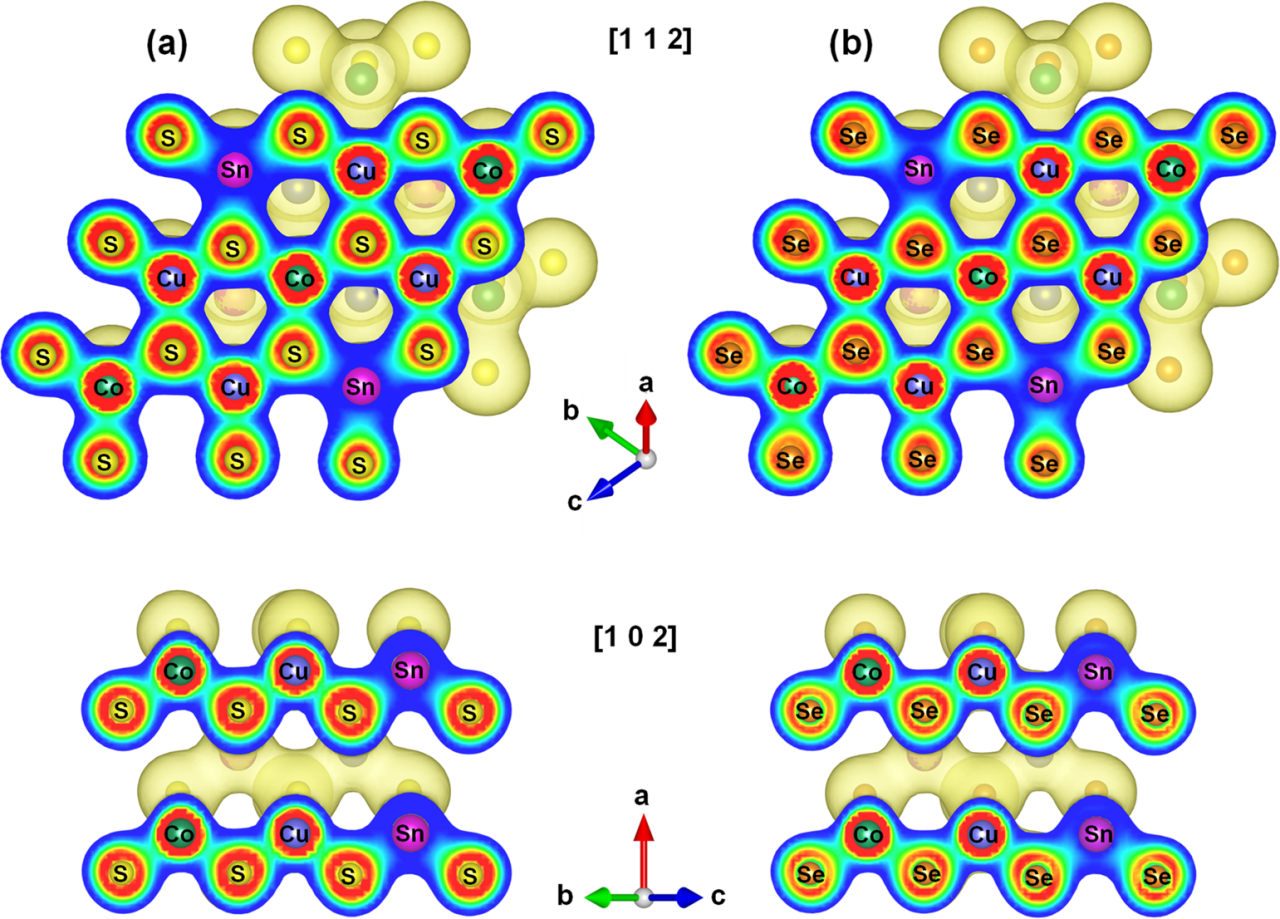
Bonding analysis in (a) Cu_2_CoSnS_4_ and (b) Cu_2_CoSnSe_4_ by means of electron localization function (ELF). The value of electron-density isosurface value is 0.02 e Bohr^–3^. 2D cross sections are going through the planes [1 1 2] and [1 0 2].

In order to understand the chemical bonding environment in Cu_2_CoSnQ_4_ (Q = S, Se), we calculated the electron localization function (ELF). The visualized 3D representation of the ELF with the 2D cross sections through the planes [1 1 2] and [1 0 2] is shown in Figure 3. This analysis indicates that the charge localized around the Co and Cu atoms strongly overlaps the charge localized around the chalcogen atoms highlighting the existence of strong covalent bonding between Co–Q and Cu–Q. In this pair of bonds, Cu–S shows slightly stronger overlapping of charge localization than Co–S in Cu_2_CoSnS_4_; however, the situation is the opposite in Cu_2_CoSnSe_4_, where Co–Se charge localization is somewhat stronger than Cu–Se. On the other hand, the weak charge localization around Sn atoms reveals a more ionic nature of Sn–Q chemical bonding. Such bonding inhomogeneity between the covalent Co–Q and Cu–Q from one side and ionic Sn–Q interactions should lead to low lattice thermal conductivity in Cu_2_CoSnQ_4_ (Q = S, Se) materials, as was also observed for other families of chalcogenides.^54−56^

**Figure 4:**
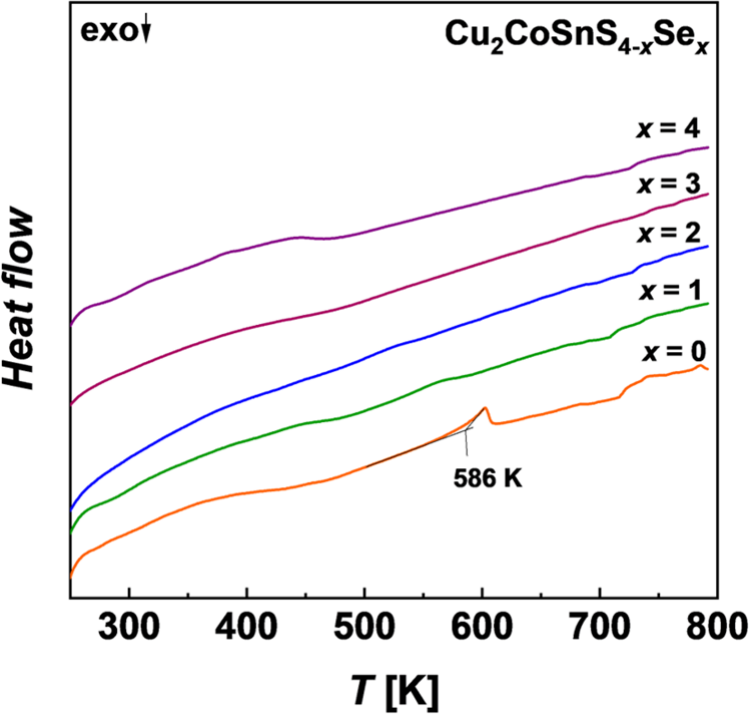
DSC curves for Cu_2_CoSnS_4–*x*_Se_*x*_ samples after synthesis.

To verify possible structural changes with the temperature, we performed Differential Scanning Calorimetry (DSC) thermal analysis (Figure 4). The DSC curve of pure sulfide shows an endothermic peak near 586 K which may indicate possible polymorphic phase transition, as was also reported for some other quaternary diamond-like compounds.^57,58^ With the increase of *x* in Cu_2_CoSnS_4–*x*_Se_*x*_ samples, this effect is almost not visible or even absent on DSC curves.

**Figure 5:**
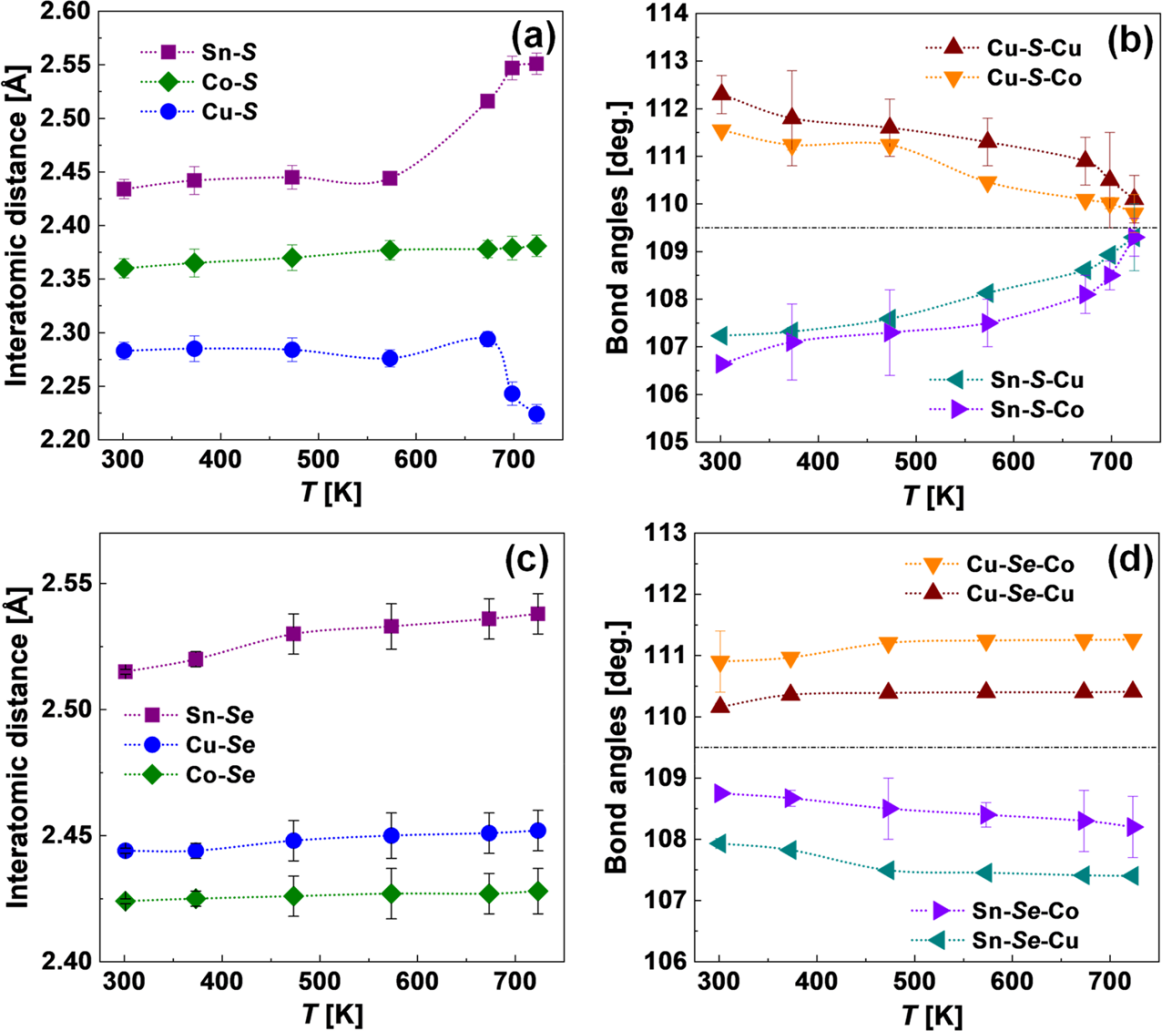
Interatomic distances (a, c), and metal–chalcogen (Q)–metal bond angles (b, d) as a function of temperature for Cu_2_CoSnS_4_ (a, b) and Cu_2_CoSnSe_4_ (c, d), respectively.

To check possible polymorphism in investigated samples, we also performed high-temperature powder XRD measurements. The resultant powder XRD patterns at different temperatures are presented in Figure S2. The Rietveld refinement was applied against all registered powder patterns. Some of the obtained structural parameters are presented in Figure 5. The interatomic distances for Cu_2_CoSnS_4_ slightly increase with temperature due to the thermal expansion of the crystal lattice (Figure 5a). However, above 600 K, the dependence of the Cu–S distance with temperature *T* shows a sharp drop down which can be attributed to the change in the Cu oxidation state from Cu^1+^ (*r* = 0.98 Å) to Cu^2+^ (*r* = 0.80 Å^53^). In turn, the Sn–S distance increases sharply above 600 K which might be related to a change in the Sn oxidation state (from Sn^4+^ (*r* = 0.67 Å) to Sn^2+^ (*r* = 1.02 Å)).^53^ These conclusions are also supported by an analysis of the dependencies of the bond angles with temperature, which is shown in Figure 5b. Particularly, the Cu–S–Cu and Cu–S–Co angles decrease while the Sn–S–Cu and Sn–S–Co angles increase with the temperature approaching the ideal tetrahedral value of 109.5° at ∼725 K (Figure 5b). The discussed changes in the interatomic distances and angles can be attributed to the phase transition of sulfide, which was observed during DSC analysis.

In the case of Cu_2_CoSnSe_4_, the modification of the crystal structure with temperature is not so large as for Cu_2_CoSnS_4_. Particularly, the interatomic distances of selenide show only a slight monotonous increase with temperature rise indicating low thermal expansion. The heating leads to an increase in Cu–Se–Cu angles and a decrease in Sn–Se–Co angles. However, the change in the bond angles is only minor and without significant fluctuations indicating an absence of the polymorphic phase transition in the Cu_2_CoSnSe_4_ compound at the analyzed temperature range. Therefore, we can conclude that the temperature dependence of the interatomic distances and bond angles are changing only slightly over the analyzed temperature range for selenide toward stable physical properties.

### 3.2 Microstructural Properties and Phase Analysis

**Figure 6:**
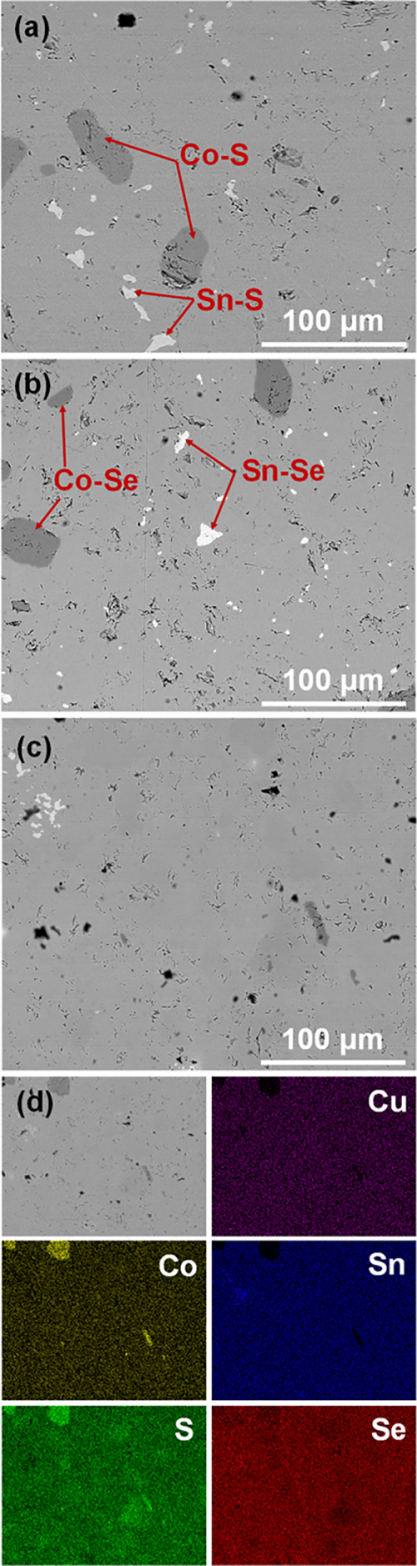
SEM images of (a) Cu_2_CoSnS_4_, (b) Cu_2_CoSnSe_4_, and (c) Cu_2_CoSnS_2_Se_2_ samples. (d) EDS elemental mapping images for the Cu_2_CoSnS_2_Se_2_ solid solution.

Figure 6 shows backscattered electron images (BSE) of selected Cu_2_CoSnS_4–*x*_Se_*x*_ samples. Except for the main phase, the presence of Co-rich regions and Sn-rich precipitates was detected. It was also noticed that solid solutions were purer than the end member compounds which can be explained by the increase of configurational entropy and consequently higher solubility of components in the system.^59,60^ According to the EDS analysis, the chemical composition of the main phase for all investigated samples was very close to the nominal composition. Co-rich regions were found to have a chemical composition close to Co_9_(S,Se)_8_ in agreement with the powder XRD data while the Sn-rich precipitates have a composition close to Sn(S,Se)_2_.

**Figure 7:**
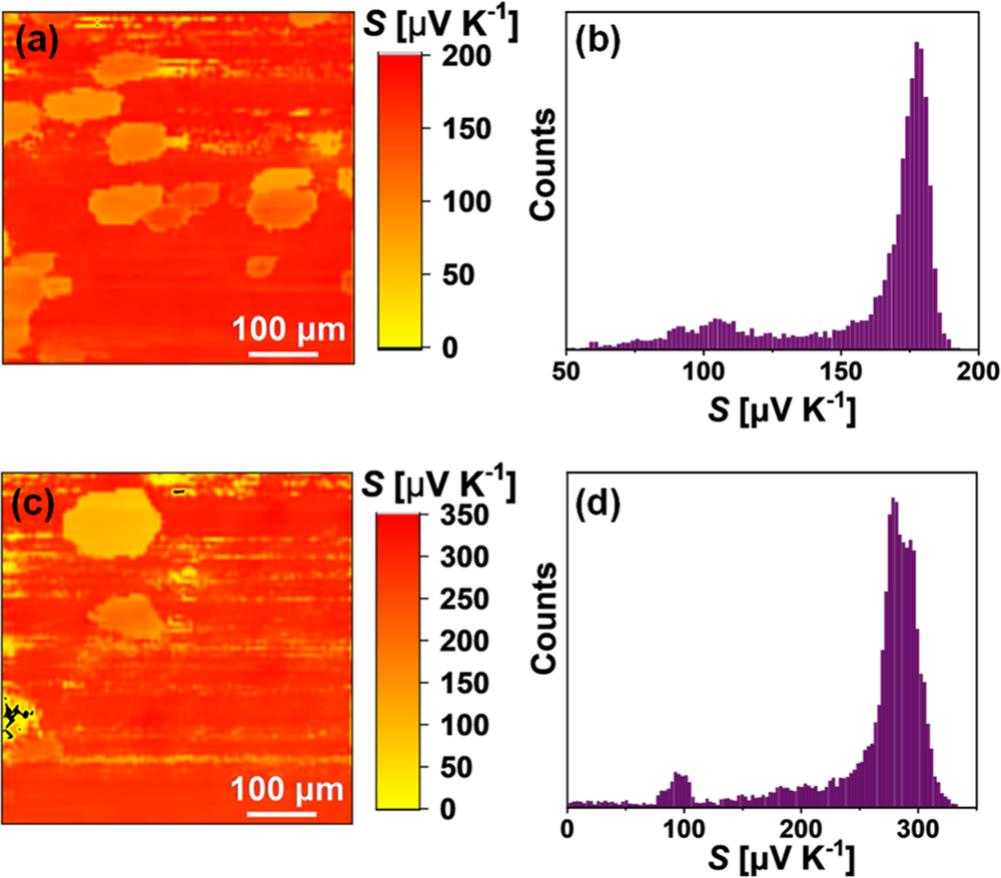
Spatial distribution (a, c) and histograms (b, d) of the Seebeck coefficient of the SPS-prepared polished surface in (a, b) Cu_2_CoSnSe_4_ and (c, d) Cu_2_CoSnS_2_Se_2_ samples.

Figure 6d shows EDS element distribution maps for the Cu_2_CoSnS_2_Se_2_ sample, where the agglomerations of Co-rich and Sn-rich phases, as well as slight inhomogeneity of the S/Se distribution from grain to grain, were detected. To check the modification of transport properties due to the presence of the impurity phases, we performed Scanning Thermoelectric Microscope (STM) measurements on the polished surface of SPS-prepared samples (Figure 7). This analysis gives the unique possibility to register the change of the Seebeck coefficient over the sample surface with a resolution of ∼1 μm. According to the Boltzmann transport theory, the Seebeck coefficient depends on the carrier concentration; hence, we can expect that the regions with the lower Seebeck coefficient have larger carrier concentrations or vice versa. In particular cases of our samples, it was registered that the grains of the precipitates have much lower values of the Seebeck coefficient indicating higher carrier concentration of the impurity phases. Therefore, the STM experiment approves the presence of highly conductive metal-like second phases. It should be mentioned that these phases may have a positive effect on the electronic transport of DLS materials, as was proposed by Zhang et al. for the case of the highly conductive CuCo_2_S_4_ second phase in Co_2.15_Co_0.8_Mn_0.05_SnS_4_.^45^

Samples for the investigation in this work were subjected to long-term annealing accompanied by the two-step powdering process (see the Experimental Details section); hence, the occurrence of the secondary phases under such conditions was unlikely. Considering this, it is of great interest to understand the thermodynamic origins of metal-like impurity phases.

**Figure 8:**
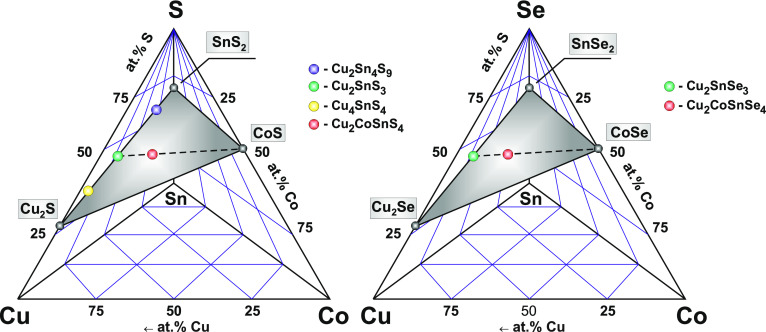
Cu_2_S(Se)–CoS(Se)–SnS(Se)_2_ sections of the Cu–Co–Sn–S(Se) ternary systems.

The quaternary chalcogenides Cu_2_CoSnS_4_ and Cu_2_CoSnSe_4_ may be considered as separate components of Cu–Co–Sn–S and Cu–Co–Sn–Se ternary systems, respectively (Figure 8). Consequently, to understand the thermodynamic condition of the formation of the quarternary chalcogenides, it is important to analyze the individual planes Cu_2_S–CoS–SnS_2_ and Cu_2_Se–CoSe–SnSe_2_. These individual planes exist as quasi-ternary systems, in which the Cu_2_CoSnS_4_ and Cu_2_CoSnSe_4_ chalcogenides are formed in the Cu_2_SnS_3_–CoS and Cu_2_SnSe_3_–CoSe quasi-binary sections, respectively.

According to ref (61), Cu_2_CoSnS_4_ is melting congruently at 1191 K, while Cu_2_CoSnSe_4_ is a peritectic compound, which is melting at 1118 K. The thermodynamic conditions of obtaining the complex chalcogenides are significantly affected by the conditions and properties of the simple phases’ formation. In accordance with refs (62 and 63), Cu_2_SnS_3_ and Cu_2_SnSe_3_ are congruent compounds and their components are insoluble. In turn, the CoS and CoSe compounds are formed at higher temperatures than Cu_2_SnS_3_ and Cu_2_SnSe_3_.^64,65^ Consequently, the presence of CoS and CoSe impurity phases in the obtained materials can be explained by the presence of a region of homogeneity of cobalt chalcogenides in binary Co–S(Se) systems. In such a condition, even very long annealing can be unhelpful in obtaining homogeneous materials.

### 3.3 Electrical and Thermal Transport Properties

**Figure 9:**
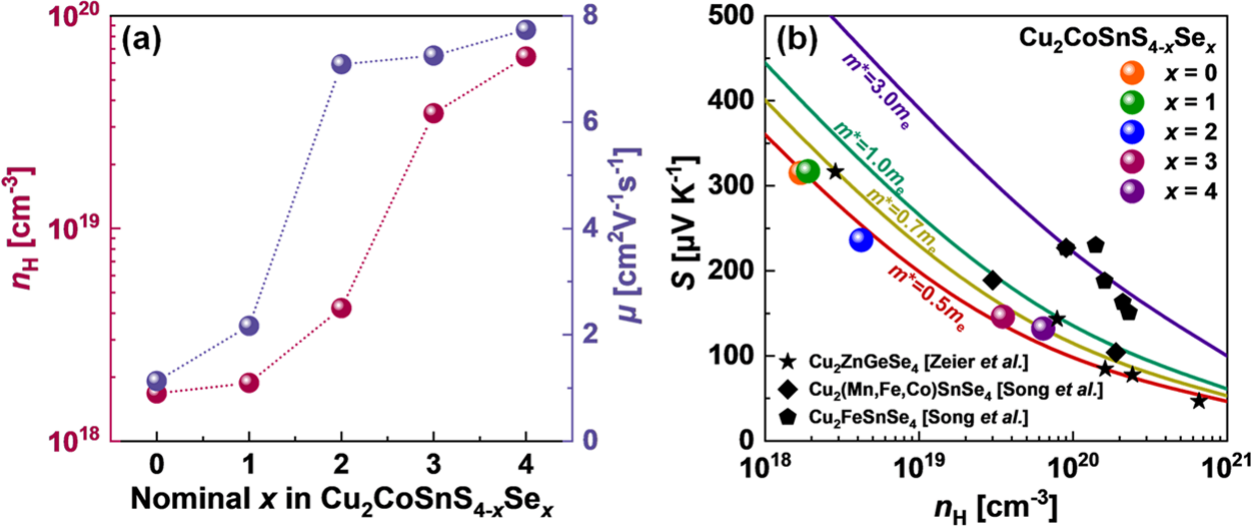
(a) Carrier concentration and mobility as a function of the nominal *x* as well as (b) the Pisarenko plot of the Seebeck coefficient and carrier concentration in Cu_2_CoSnS_4–*x*_Se_*x*_. Dashed lines are shown as a guide for the eye, while solid lines were calculated within the Kane band model.

Transport properties for the investigated materials at *T* = 298 K are shown in Table 2. The Seebeck coefficient *S* and electrical resistivity ρ in Cu_2_CoSnS_4–*x*_Se_*x*_ solid solutions decrease with the increase of Se content mainly due to the increase of the carrier concentration *n*_H_. Although Se is an isovalent dopant in Cu_2_CoSnS_4–*x*_Se_*x*_, we registered a growth of charge carrier concentration from 1.7 × 10^18^ to 6.4 × 10^19^ cm^–3^ (Figure 9a) in the S → Se direction, which may be provoked by the increase of intrinsic point defects concentration. A similar effect was also observed in the work of Zhao et al.,^66^ where the authors have shown that the alloying of S at Se sites increases the bonding energy in Cu_2_Se_1–*x*_S_*x*_ materials restricting the formation of Cu vacancies. This resulted in a much lower carrier concentration in Cu_2_S compared to Cu_2_Se. Hence, we may hypothesize that, in our case of the Cu_2_CoSnS_4–*x*_Se_*x*_ materials, the increase of the carrier concentration with an *x* increase is also connected with the decrease of the bonding energy, i.e., weakening of Me–Q bonds.

**Table 2:** Seebeck Coefficient *S*, Electrical Resistivity ρ, Thermal Conductivity κ, Hall Carrier Concentration *n*_H_, Carrier Mobility μ, and Effective Mass m* Calculated Using the Single Kane Band (SBK) and Single Parabolic Band (SPB) Models for the Cu_2_CoSnS_4–*x*_Se_*x*_ Polycrystalline Samples at *T* = 298 K

Cu_2_CoSnS_4–*x*_Se_*x*_	*S* [μV K^–1^]	ρ [Ω cm]	κ [W m^–1^ K^–1^]	*n*_H_ [cm^–3^]	μ [cm^2^ V^–1^ s^–1^]	m*/m (SKB)	m*/*m* (SPB)
*x* = 0	315	3.3	4.2	1.7 × 10^18^	1.1	0.5	0.47
*x* = 1	317	1.5	2.7	1.9 × 10^18^	2.2	0.55	0.52
*x* = 2	236	0.21	2.2	4.2 × 10^18^	7.1	0.46	0.42
*x* = 3	146	0.025	1.8	3.5 × 10^19^	7.3	0.69	0.6
*x* = 4	132	0.013	1.6	6.4 × 10^19^	7.7	0.84	0.72

The carrier mobility of the investigated alloys is in the range of 1.1–7.7 cm^2^ V^–1^ s^–1^, which is comparable with the μ values previously reported for Cu-contained diamond-like structure sulfides and selenides.^40,44^ Intriguingly, with the increase of the Se content in Cu_2_CoSnS_4–*x*_Se_*x*_ specimens, a rise of carrier concentration is observed simultaneously with the increase of mobility, which is not agreed with the classical electronic transport behavior in semiconductor materials. This observation can be connected with the different bandgaps; however, as the reported bandgap values for sulfide and selenide are not very different (around 1.2–1.8 eV for Cu_2_CoSnS_4_^45^ and 1–1.5 eV for Cu_2_CoSnSe_4_^44^ at 298 K), it looks to be unlikely. A more probable cause for the simultaneous increase of carrier concentration and mobility is connected with the crystal structure differences between selenide and sulfide. Particularly, in the S → Se direction of Cu_2_CoSnS_4–*x*_Se_*x*_ solid solutions, all angles in the structure are approaching the ideal tetrahedral value of 109.5°, which is an indicator of the higher symmetry of the structure (see the Crystal Structure and Bonding Analysis section). In turn, the higher symmetry of selenide promotes the higher mobility μ of this material. Therefore, the carrier mobility increases with the increase of the Se content in Cu_2_CoSnS_4–*x*_Se_*x*_ solid solutions. A similar enhancement of carrier mobility was also recorded in the work of Song et al.^44^ Particularly, in the case of Cu_2_MnSnSe_4_, Cu_2_FeSnSe_4_, and Cu_2_CoSnSe_4_ DLS materials investigated by the authors, the room temperature electronic transport properties are becoming better from Cu_2_MnSnSe_4_ to Cu_2_CoSnSe_4_ with the approach of the interatomic bond angles to the ideal tetrahedral value of 109.5°. Moreover, the shown carrier mobility of Cu_2_CoSnSe_4_ is very similar to the carrier mobility of Cu_2_MnSnSe_4_, even if the carrier concentration changes significantly.^44^ These results well agreed with the proposed statement about the influence of crystal symmetry on carrier mobility.

The effect of crystal symmetry on carrier mobility is also connected with the type of bonding. From the chemical point of view, heteroatomic bonds lead to increased electron scattering and degrade carrier mobility, while homoatomic bonds cause good carrier mobility.^54^ High mobility can be achieved even in materials with heteroatomic bonds; however, a high crystal symmetry and a small difference in electronegativity are necessary. A simple indicator of the existence of these factors in the investigated DLS material is the ionicity of bonds. For the Me–Se bonds, the ionicity is lower compared to the Me–S bonds and, consequently, the carrier mobility of Cu_2_CoSnSe_4_ is higher compared to that of Cu_2_CoSnS_4_.

The Pisarenko plot of the Seebeck coefficient as a function of the carrier concentration is shown in Figure 9b. As the carrier concentrations for the investigated alloys are in the range of weak and heavy degenerated statistics, we perform the calculations of the effective masses considering both parabolic and Kane band models.^67,68^ The results of m* calculations show only a small difference between the effective mass calculated using these two approximations, indicating their acceptability for the case of our samples (Table 2). During the calculations, we consider the acoustic phonon scattering (*r* = 0) as the main scattering mechanism. All details of the performed calculations can be found in the Supporting Information.^12,18,60^ The plotted dependence of *S*(*n*_H_) is in agreement with the previously published data for Cu_2_ZnGeSe_4_ and Cu_2_(Mn,Co)SnSe_4_.^40,44,69^ The effective masses, which show the best fitting between the experimental data and theoretical curves, are in the range of 0.5–1.0 *m*_e_. This result agrees with the DOS effective masses m* obtained for Cu_2_ZnGeSe_4_ and Cu_2_(Mn,Co)SnSe_4_. However, the estimated values of m* for Cu_2_CoSnS_4–*x*_Se_*x*_ are much lower than those of Cu_2_FeSnSe_4_, where m* ∼ 3.0 *m*_e_, as reported by Song et al.^44^ This observation may indicate a large difference in band structures for Fe-contained stannite compounds or some other effects related to the nature of the Seebeck coefficient, electrical conductivity, or Hall constant.

**Figure 10:**
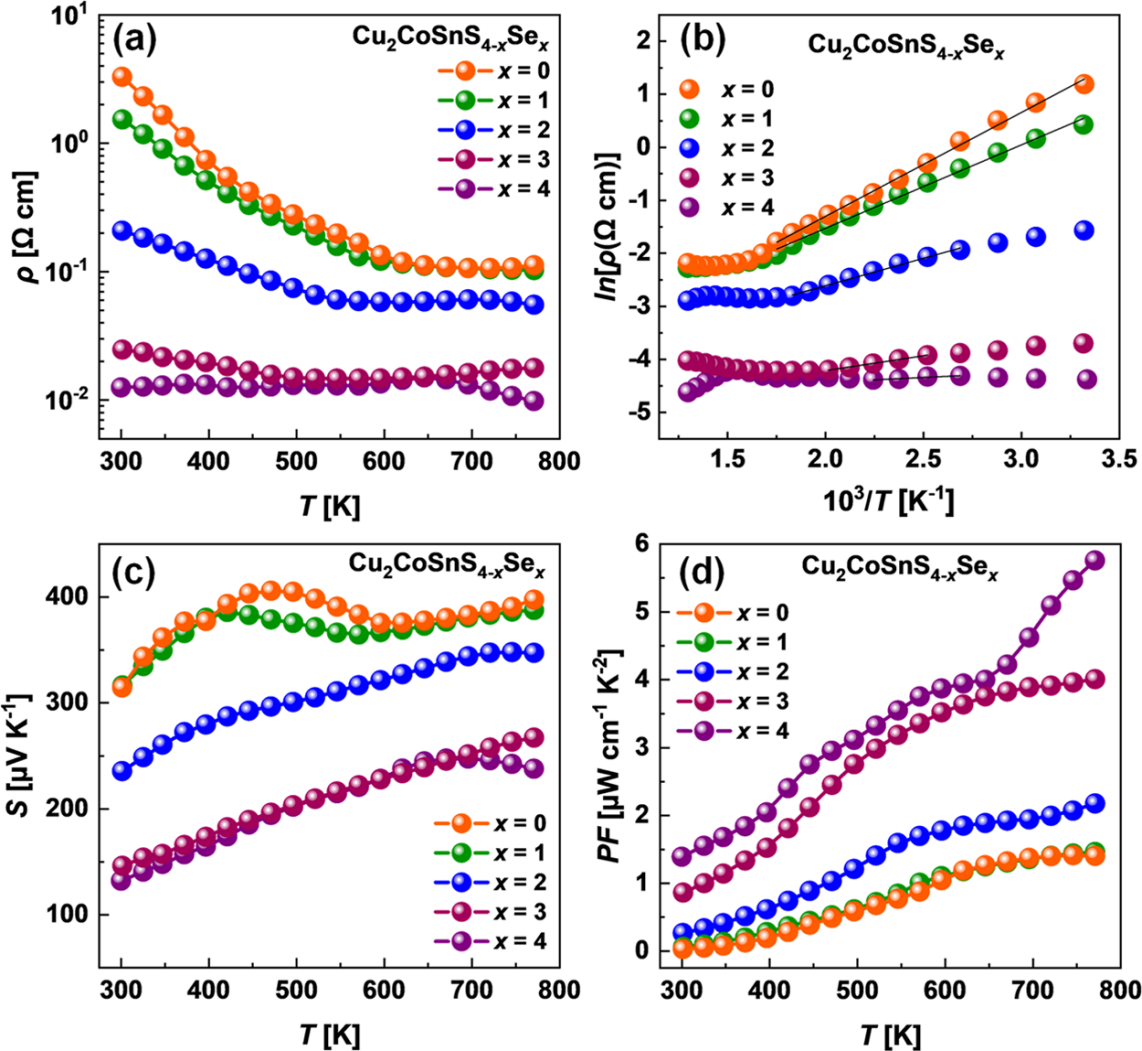
(a) Electrical resistivity, (b) Arrhenius plot of electrical resistivity, (c) Seebeck coefficient, and (d) thermoelectric power factor *PF* for Cu_2_CoSnS_4–*x*_Se_*x*_ materials.

Figure 10 shows the electrical resistivity (panel a) and Arrhenius plot of electrical resistivity (panel b) for Cu_2_CoSnS_4–*x*_Se_*x*_ polycrystalline samples over the entire temperature range of 298–773 K. As expected for the undoped semiconductors with relatively large bandgaps, all sulfur-contained samples investigated in this work show high resistivity with decreasing temperature trends. In turn, the electrical resistance of pure selenide is lower and even shows a slight increasing trend, indicating a metal-like type of electrical transport. This observation can be well explained by the relatively high carrier concentration and Hall mobility, which were measured for selenide. Around the temperature of phase transition, the change of the slope of the ρ(*T*) dependences can be observed for sulfur-contained samples. Particularly, below phase transition, as is expected for the intrinsic semiconductors, one can indicate the sharp decreasing dependence of ρ(*T*). Above phase transition, the electrical resistance is approaching the constant value or slightly increasing, which suggests the existence of a metal–insulator transition.^57^ We also should mention that minor fluctuations in electrical resistance can be connected with the highly conductive impurity phases, which were detected in our samples. The effect of these phases on the value of ρ(*T*) is expected, especially in the high-temperature range, as the higher energies make it possible for the carriers to overcome the interface potential barriers.

The activation energies *E*_a_ estimated from the Arrhenius plot of electrical resistivity (Figure 10b) for Cu_2_CoSnS_4–*x*_Se_*x*_ polycrystalline samples are given in Table 3. The estimated activation energies decrease from 0.34 to 0.03 eV with the increase of Se content *x* in Cu_2_CoSnS_4–*x*_Se_*x*_. The values of *E*_a_ are lower than the reported bandgaps for pure selenide and sulfide and might suggest the presence of some in-gap states, as it was recently shown for the cases of Cu_0.8+*y*_Ag_0.2_In_1–*y*_Te_2_^70^ and (Cu_1–*x*_Ag_*x*_)(In_1–*y*_Ga_*y*_)Te_2_^71^ DLS materials.

**Table 3:** Activation Energy *E*_a_ in the Given Temperature Range for Studied Cu_2_CoSnS_4–*x*_Se_*x*_ Diamond-Like Compounds

Cu_2_CoSnS_4–*x*_Se_*x*_	*E*_a_ [eV]	temp. range [K]
*x* = 0	0.34	298–573
*x* = 1	0.27	298–573
*x* = 2	0.18	373–548
*x* = 3	0.10	398–498
*x* = 4	0.03	373–448

Figure 10c shows the Seebeck coefficient as a function of temperature for the investigated Cu_2_CoSnS_4–*x*_Se_*x*_ diamond-like compounds. The values of *S* are positive at the investigated temperature range, indicating holes as the dominative charge carriers. While the phase transition has an obvious effect on the *S*(*T*) trend for sulfide, the Seebeck coefficient for selenide shows a monotonous increase with temperature up to the high-temperature range. Considering the large bandgap for the investigated alloys, we do not expect that a slight decrease of *S*(*T*) for Cu_2_CoSnSe_4_ at high temperatures is connected with the excitation of the minority carriers. This effect may be related to the participation of the highly conductive second phase observed in our samples.

Combining the measured Seebeck coefficient *S* and electrical resistivity ρ, the thermoelectric power factor (*PF* = *S*^2^ρ^–1^) was evaluated for all investigated samples (Figure 10d). The temperature dependencies of the *PF* show a positive increasing trend over the investigated temperature range. The high carrier concentration of 6.4 × 10^19^ cm^–3^ recorded for selenide leads to the highest power factor of up to 6.0 μW cm^–1^ K^–2^ at 773 K in this compound, which is comparable with the best reported diamond-like structure selenides.^35^

**Figure 11:**
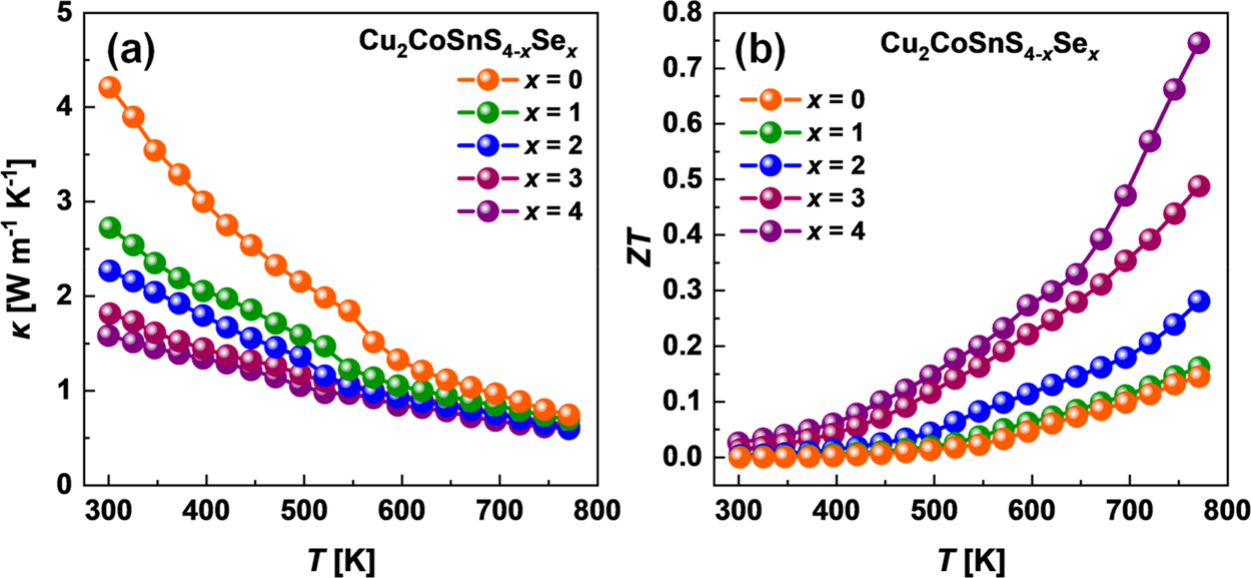
(a) Thermal conductivity and (b) dimensionless thermoelectric figure of merit as a function of temperature for Cu_2_CoSnS_4–*x*_Se_*x*_ materials.

The temperature-dependent total thermal conductivity κ for Cu_2_CoSnS_4–*x*_Se_*x*_ solid solutions is shown in Figure 11a. The thermal conductivity decreases with a temperature approaching very low values below ∼0.7 W m^–1^ K^–1^ at 773 K for all investigated samples. The possible reasons for such a significant reduction of the total thermal conductivity in the investigated diamond-like Cu_2_CoSnS_4–*x*_Se_*x*_ solid solutions will be discussed in the following section. Due to the large bandgap, we do not observe the effect of the minority carrier excitation, which can be detected by bipolar conduction. If the effect of the phase transition can be observed at around 550 K for sulfide, it was not detected in selenide.

To evaluate the energy conversion performance of the investigated materials, we have calculated the dimensionless thermoelectric figure of merit *ZT*, which is shown in Figure 11b. Thanks to the highest power factor and the lowest total thermal conductivity, the *ZT* trend for undoped selenide is the highest and reaches a maximum value of ∼0.75 at 773 K. In turn, the thermoelectric performance of this material can be further improved through carrier concentration optimization. The heating/cooling cycles of the Seebeck coefficient, electrical resistivity, thermal conductivity, and *ZT* parameter for the investigated Cu_2_CoSnS_4–*x*_Se_*x*_ materials are shown in Figures S3 and S4. In contrast to many other Cu-based sulfides and selenides,^60,72^ the investigated DLS materials show good repeatability of the thermoelectric properties (Figure S3a–d).

### 3.4 Origins of the Low Lattice Thermal Conductivity

**Figure 12:**
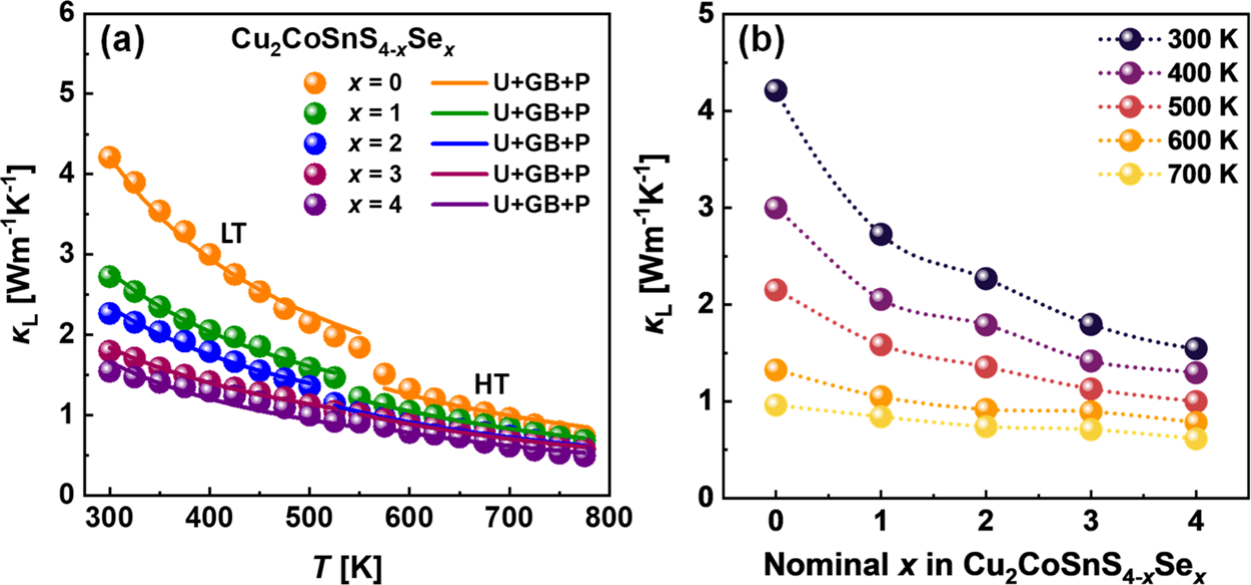
(a) Lattice thermal conductivity for the studied Cu_2_CoSnS_4–*x*_Se_*x*_ materials, lines correspond to the calculations using the Debye-Callaway approach. Here, U, GB, and P represent Umklapp scattering, grain boundary scattering, and point defect scattering, respectively. The low-temperature (LT) region was analyzed using the three-phonon Umklapp process, while the high-temperature (HT) region was fitted using the four-phonon Umklapp process. (b) κ_L_ as a function of *x* in studied Cu_2_CoSnS_4–*x*_Se_*x*_ materials at selected temperatures.

The total thermal conductivity κ of the semiconductor materials can be estimated as a sum of the lattice κ_L_, electronic κ_e_, and bipolar components κ_b_ (κ = κ_L_ + κ_e_ + κ_b_). In the case of relatively wide-bandgap materials, the bipolar component of the thermal conductivity can be neglected. To estimate the electronic contribution to the thermal conductivity, we used the Wiedemann–Franz law (κ_e_ = *LT*ρ^–1^, where *L* is the Lorenz number). The lattice thermal conductivity κ_L_ was determined by subtracting κ_e_ from κ. The temperature-dependent Lorenz number as a function of temperature was calculated considering the Kane band model approximation using the following expression:
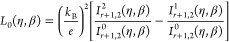
1where *k*_B_, *e*, and *r* denote the Boltzmann constant, charge of the electron, and scattering parameter, respectively. The calculated lattice thermal conductivity as a function of temperature is shown in Figure 12a. Due to the large electrical resistivity, the total thermal conductivity of the investigated alloys mainly consists of the lattice contribution κ_L_.

To illustrate the effect of the anion substitution on the thermal transport of the Cu_2_CoSnS_4–*x*_Se_*x*_ alloys, we performed ultrasonic measurements and combined the obtained results with the Debye-Callaway approach calculations. The measured values of the longitudinal *v*_l_ and transverse *v*_t_ speed of sound accompanied by the estimated values of Debye temperatures Θ_D_, Bulk modulus *B*, Young modulus *E*, the Poisson ratio ν, Grüneisen parameter γ, phonon mean free path *l*_ph_, and the minimum thermal conductivity κ_glass_ for Cu_2_CoSnS_4–*x*_Se_*x*_ alloys are shown in Table 4. All details about the determination of the elastic and thermal transport properties can be found in eqs S4–S13.

**Table 4:** Elastic and Thermal Transport Properties of Cu_2_CoSnS_4–*x*_Se_*x*_ Alloys

Cu_2_CoSnS_4–*x*_Se_*x*_	*v*_l_, m s^–1^	*v*_t_, m s^–1^	*v*_m_, m s^–1^	Θ_D_, K	ν	γ	*B*, GPa	*E*, GPa	*l*_ph_, Å	κ_glass_, W m^–1^ K^–1^
*x* = 0	4398	2289	2562	276.4	0.31	1.86	52.9	58.9	18.5	0.69
*x* = 1	4386	2243	2512	270.1	0.32	1.93	58.8	62.4	13.6	0.66
*x* = 2	4314	2229	2495	267.6	0.32	1.89	61.3	66.9	12.4	0.64
*x* = 3	3713	1991	2223	232.9	0.30	1.76	44.1	53.4	18.2	0.55
*x* = 4	3754	1991	2226	236.0	0.30	1.80	51.2	60.2	11.3	0.53

Although the Grüneisen parameters are close for the investigated samples, the average speed of sound in the investigated Cu_2_CoSnS_4–*x*_Se_*x*_ alloys decreases from 2562 to 2226 m s^–1^ with the increase of Se content, in accordance with the decrease of the lattice thermal conductivity. This observation can be caused by the weakening of chemical bonds in the S → Se direction. If the longitudinal speed of sound shows relatively high values (3754 m s^–1^ for selenide and 4398 m s^–1^ for sulfide), the transverse speed of sound is rather moderated or even low (Table 4). This contributes to much higher values of the Grüneisen parameters γ ∼ 1.8–1.9 for the investigated quaternary DLS materials compared to the binary DLS compounds, which usually show γ ∼ 0.5–0.7.^73^ As the Grüneisen parameter can be defined as a change of the phonon frequency with atomic volume, the large lattice anharmonicity and low lattice thermal conductivity for these alloys can be expected. The large anharmonic vibration in the crystal lattice is expected in materials with large atomic coordination numbers, rattling atoms, or bonding inhomogeneity.^73^ As the large atomic coordination numbers and rattling atoms are not inherent for the investigated DLS materials, the bonding inhomogeneity caused by the different polarity of Me–Q bonds is the most probable reason for the large anharmonicity. Moreover, the bonding inhomogeneity, which is reflected in the different bond lengths and bond angle variations, may change the local bulk modulus. Such changes, in addition to mass field fluctuations, will increase the strain field and hence the scattering by point defects.^73^ The values of κ_L_ of Cu_2_CoSnS_4–*x*_Se_*x*_ samples at elevated temperatures approach the glass limit of the lattice thermal conductivity, described by Cahill et al.,^74^ assuming a minimum scattering length for phonons as a function of the phonon frequencies.

To evaluate the dominant phonon scattering mechanism on the lattice thermal conductivity of the investigated Cu_2_CoSnS_4–*x*_Se_*x*_ alloys, we employed the Debye-Callaway approach.^75^ Within this model, the lattice thermal conductivity can be approximately estimated as follows:
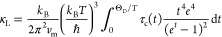
2where τ_c_ is the total phonon relaxation time, which can be expressed using Matthiessen’s rule:
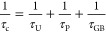
3Here, τ_U_, τ_P_, and τ_GB_ denote the phonon relaxation time contributed from the phonon–phonon Umklapp processes scattering, point defect scattering, and grain boundary scattering, respectively (eqs 4–6).
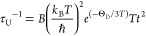
4
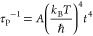
5
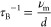
6where ℏ = *h*/(2π), *t* = ℏω/(*k*_B_*T*), *d* is the grain size, *A* and *B* are the fitting constants that represent the point-defect scattering and three-phonon Umklapp scattering, respectively. All temperature trends of κ_L_ were reasonably well fitted over the temperature range of 300–550 K (Figure 12a), which corresponds to the low-temperature modification. As the grain boundary scattering is expected to be very similar for all investigated samples, our further analysis was focused on the fitting parameters *A* and *B*. As can be seen in Table 5, parameter *A* shows higher values for the case of the Cu_2_CoSnS_4–*x*_Se_*x*_ samples with *x* = 2 and 3, which is connected to stronger point defect scattering in these materials. In turn, parameter *B* increases from sulfide to selenide, indicating significant enhancement of phonon–phonon Umklapp scattering in the S → Se direction and suggesting even larger lattice anharmonicity in selenide compared to sulfide. The lower speed of sound and phonon mean a free path for selenide, also well correlating with the hypothesis about the larger lattice anharmonicity in this material (Table 5).

**Table 5:** Fitting Parameters *A* and *B*, Which Have Been Used for the Calculation of Lattice Thermal Conductivity by the Debya-Callaway Approach

	low-temperature phase		high-temperature phase	
Cu_2_CoSnS_4–*x*_Se_*x*_	*A* × 10^42^, s^3^	*B* × 10^17^, s^3^ K^–2^	*A*, s^3^	*B*_1_ × 10^45^, s^3^ K^–2^
*x* = 0	-	1.04		1.41
*x* = 1	1.54	1.32		1.85
*x* = 2	2.83	1.43		2.25
*x* = 3	6.69	1.56		2.82
*x* = 4	1.72	2.28		3.36

In the high-temperature region (>550 K), the employment of the three-phonon Umklapp, point defect, and grain boundary scattering does not give an acceptable agreement between the theoretical and experimental lattice thermal conductivity. The reasonable fitting agreement with the experimental points was obtained by employing the four-phonon Umklapp scattering, which is highly probable for the systems with high lattice anharmonicity. The phonon relaxation time contributed to the four-phonon Umklapp scattering can be estimated as follows:^76^
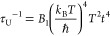
7where *B*_1_ is the fitting constant that represents the four-phonon Umklapp scattering. As can be seen in Figure 12a, good agreement with the experimental κ_L_ was achieved by the implementation of only four-phonon Umklapp and grain boundary scattering. Parameter *B*_1_ increases with an *x* rise in Cu_2_CoSnS_4–*x*_Se_*x*_, indicating the intensification of the lattice anharmonicity in the S → Se direction.

Figure 12b shows the lattice thermal conductivity as a function of Se content *x* in studied Cu_2_CoSnS_4–*x*_Se_*x*_ materials at selected temperatures. It should be mentioned that the κ_L_(*x*) trends decrease in the whole measured temperature range, which contradicts the expected valley-like trend due to mass-strain fluctuation. The quite sharp decrease of the lattice thermal conductivity from sulfide to selenide can be explained by several simultaneous effects. Particularly, the enhancement of the phonon scattering in the investigated DLS materials is expected due to (i) the weakening of interatomic interactions between cations and anions, as it is shown in the crystal structure section; (ii) the increase of a number of point defects reflected in a significant rise of the carrier concentration; (iii) heavier Se atoms, which decrease the frequency of lattice vibrations; (iv) strengthening of phonon–phonon Umklapp scattering in the S → Se direction as was derived from the Callaway modeling. Although some other effects, e.g., scattering on grain boundaries or interphases, influence the κ_L_ values, these factors should be of the same order for all investigated samples. The decreasing κ_L_(*x*) tendencies also supported the above discussion about the lattice anharmonicity as the origin of the low thermal conductivity for the investigated DLS materials.

## 4 Conclusions

In summary, we have investigated the effect of the anion substitution on the structural, microstructural, and thermoelectric properties of the Cu_2_CoSnS_4–*x*_Se_*x*_ alloys. The performed investigations revealed mainly the single-phase nature of the investigated samples. Only minor highly conductive impurity phases with a composition close to Co_9_(S,Se)_8_ and Sn(S,Se)_2_ were detected. The thermal analysis shows the possible phase transition for sulfide at 586 K, while this thermal effect is much lower or absent in the rest of the investigated samples.

Unexpectedly, the Hall concentration *n*_H_ and mobility μ increase simultaneously with the rise of *x* in Cu_2_CoSnS_4–*x*_Se_*x*_. The increase in the concentration is connected with the decrease of the activation energy *E*_a_, while the enhancement of the mobility can be attributed to the higher crystal symmetry of Se-rich samples. As a result, the electrical resistivity for selenide is much lower than for sulfide. Due to the large difference in the carrier concentration (1.7 × 10^18^ to 6.4 × 10^19^ cm^–3^), the Seebeck coefficient is also changed significantly in the Cu_2_CoSnS_4*–x*_Se_*x*_ series from 132 μV K^–1^ for selenide up to 315 μV K^–1^ for sulfide. The thermal conductivities for the investigated DLS materials are in the range of 1.6–4.2 W m^–1^ K^–1^ at 298 K, while the lowest κ values (below ∼0.7 W m^–1^ K^–1^) were recorded at 773 K. Such low values of the thermal conductivity are mainly attributed to the bonding inhomogeneity between the three types of cation and anion atoms. As it was evaluated from the ultrasonic measurements combined with the Debye-Callaway calculations, the lattice thermal conductivity for selenide is much lower than that for sulfide, mainly due to the larger anharmonicity.

As a result of the largest power factor and the lowest thermal conductivity, the thermoelectric figure of merit *ZT* reaches the maximum value of 0.75 at 773 K for Cu_2_CoSnSe_4_. This study highlights the possibility of how to effectively modify the electronic and thermal transport properties of the DLS materials. Particularly, high carrier mobility can be obtained through the promotion of crystal symmetry, while lattice thermal conductivity can be effectively suppressed through the bonding inhomogeneity and lattice anharmonicity approaches.

## Supporting Information Available

Rietveld refinements; lattice parameters; high-temperature powder XRD patterns; heating and cooling data of thermoelectric properties; details of the Lorenz number calculations; details of the elastic and thermal transport properties calculations (PDF)
