# Supplementary material for: Structure Evolution and Bonding Inhomogeneity toward High Thermoelectric Performance in Cu2CoSnS4–xSex Materials
Source: Chem Mater. 2023 Jun 7;35(12):4772–85. doi: 10.1021/acs.chemmater.3c00586 (PMC10311630; doi:10.1021/acs.chemmater.3c00586)
Supplement: Supplementary file 1 — cm3c00586_si_001.pdf [file cm3c00586_si_001.pdf]

Supporting Information

Structure evolution and bonding inhomogeneity towards high thermoelectric performance in $Cu_2CoSnS_{4-x}Se_x$ materials

Taras Parashchuk[1*], Oleksandr Cherniushok[1], Oleksandr Smitiukh[2], Oleg Marchuk[2], and Krzysztof T. Wojciechowski[1*]

[1]Thermoelectric Research Laboratory, Department of Inorganic Chemistry, Faculty of Materials Science and Ceramics, AGH University of Science and Technology, Mickiewicza Ave. 30, 30-059 Krakow, Poland
[2]Department of Chemistry and Technology, Volyn National University, Voli Ave 13, Lutsk, 43025, Ukraine

*Corresponding author(s):

Taras Parashchuk, E-mail: parashchuk@agh.edu.pl.

Krzystof T. Wojciechowski, E-mail: wojciech@agh.edu.pl

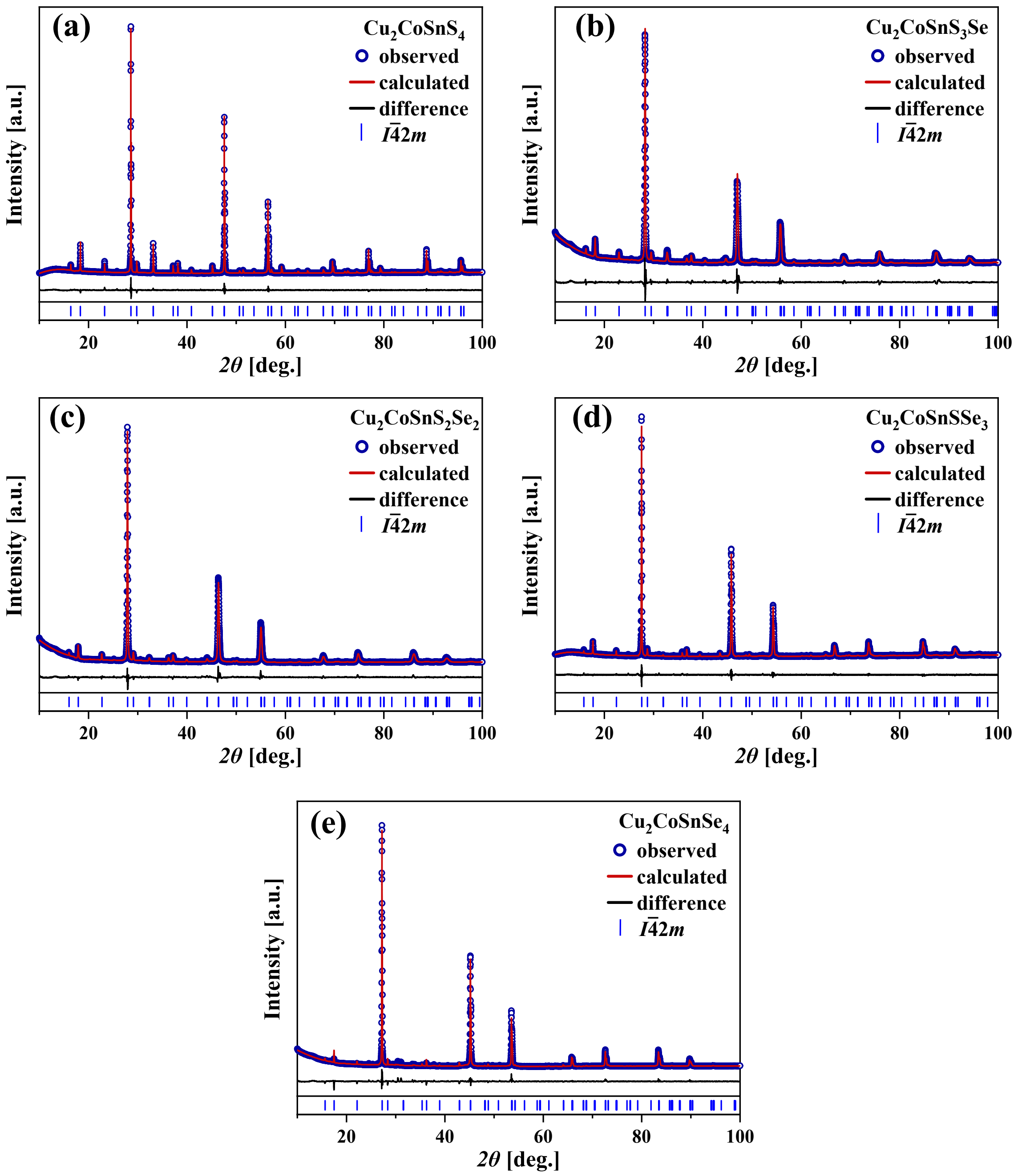

Figure S1. Results of the Rietveld refinement for $Cu_2CoSnS_{4-x}Se_x$ samples.

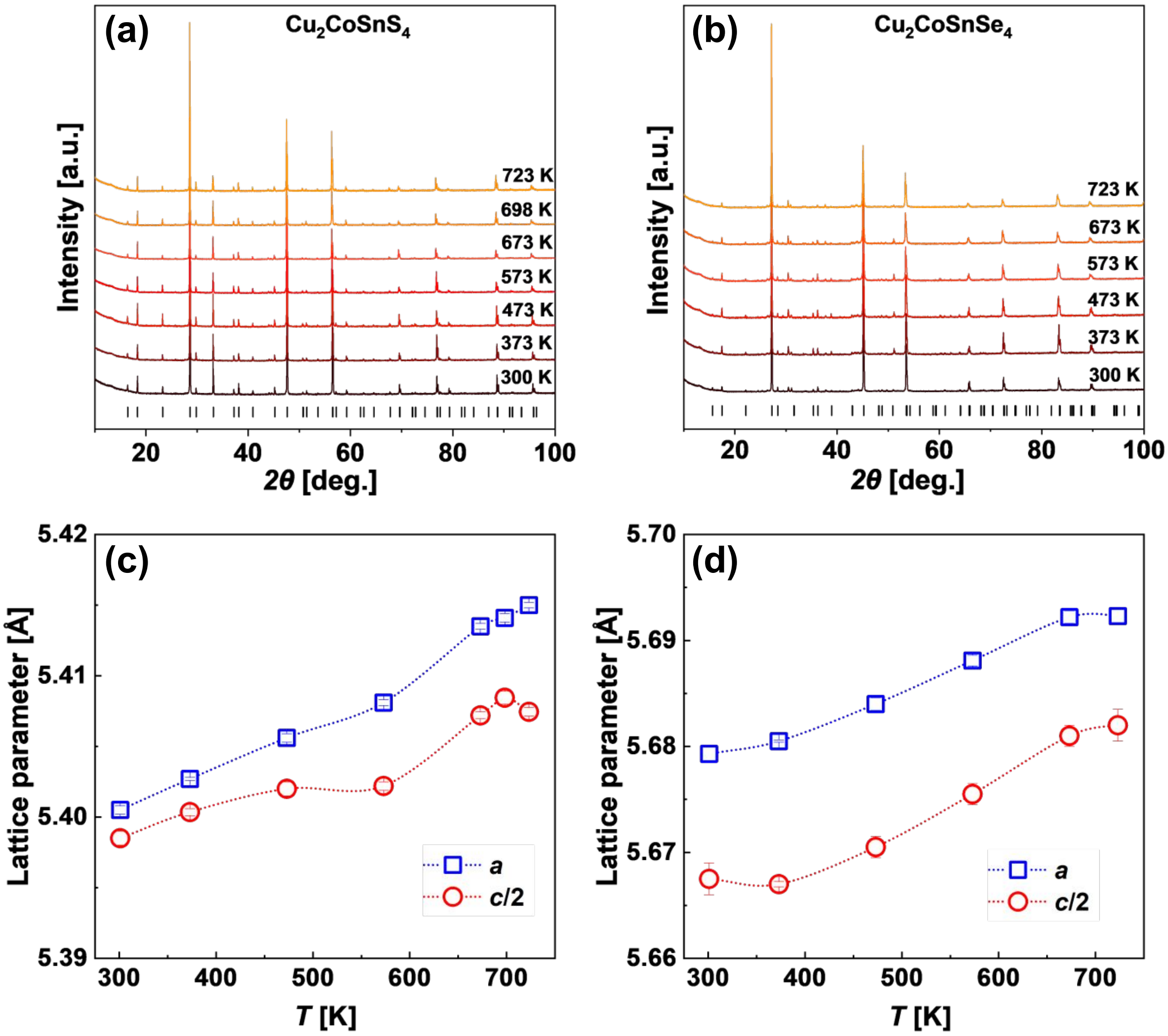

Figure S2. High-temperature powder XRD patterns of (a) Cu_2CoSnS_4 and (b) $Cu_2CoSnSe_4$ samples and corresponding changes in lattice parameters for (c) Cu_2CoSnS_4 and (d) $Cu_2CoSnSe_4$.

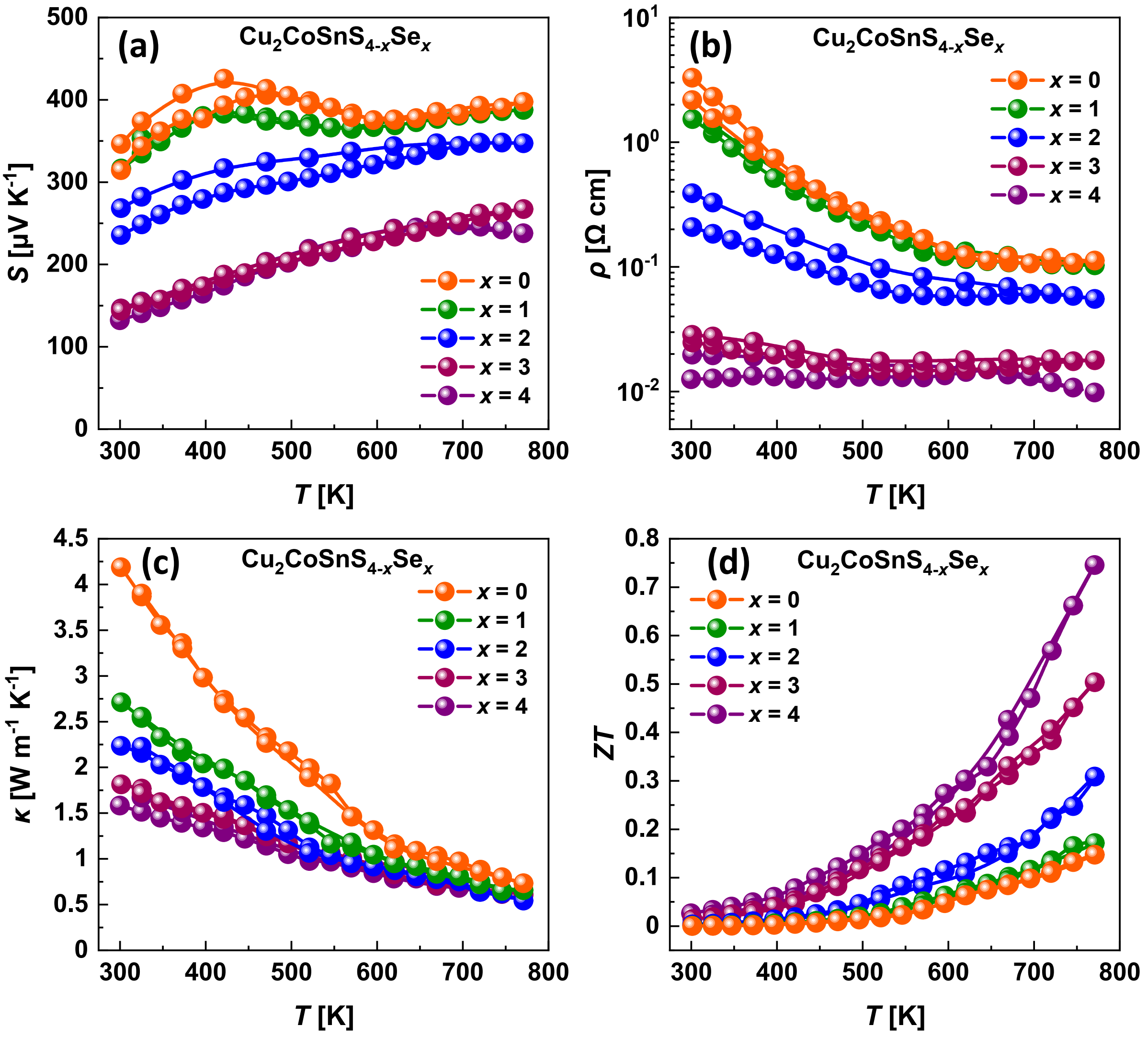

Figure S3. Heating and cooling cycle of (a) Seebeck coefficient, (b) electrical resistivity, (c) thermal conductivity, and (d) *ZT* parameter for $Cu_2CoSnS_{4-x}Se_x$ materials.

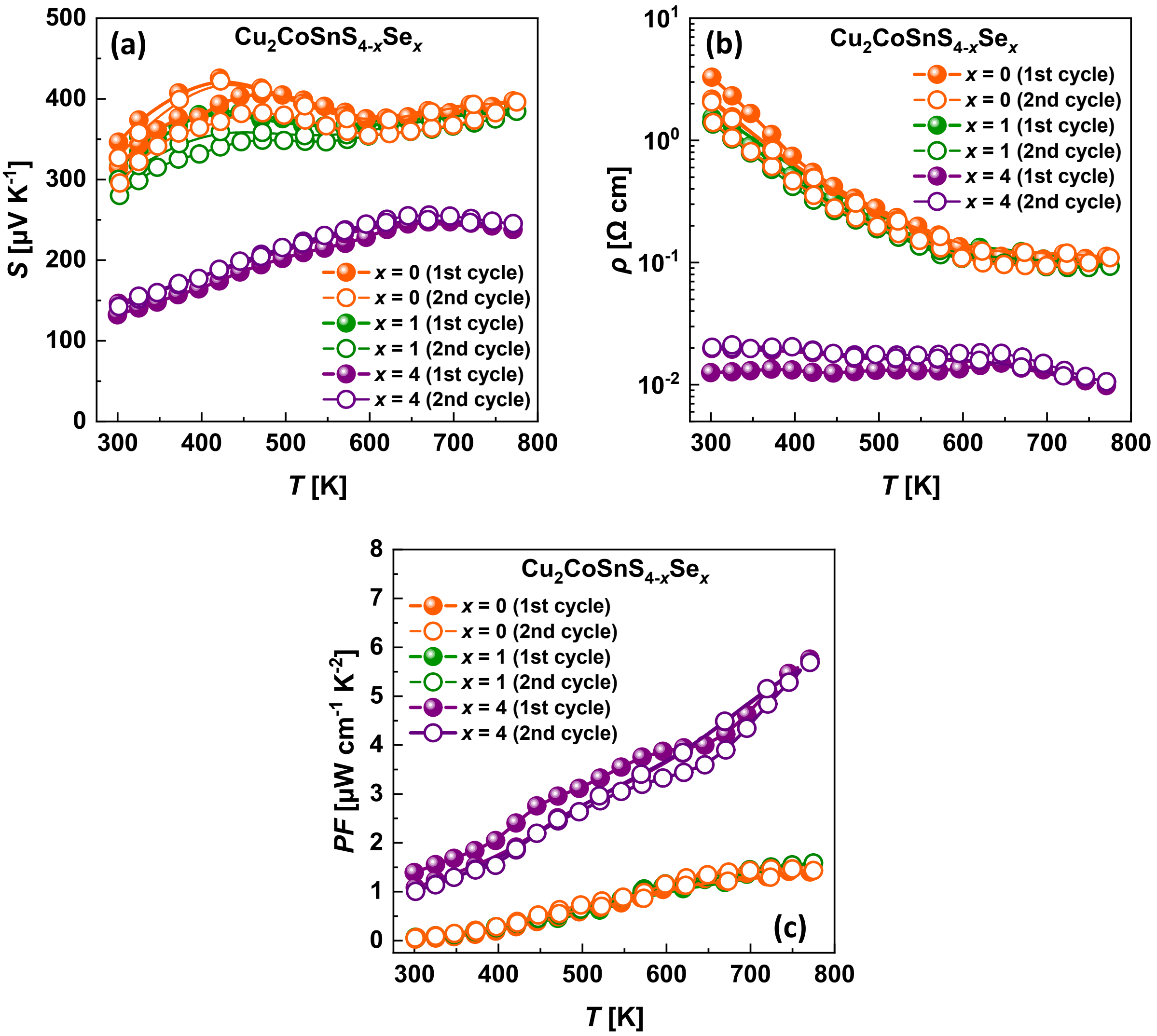

Figure 4. Cycling tests of (a) Seebeck coefficient, (b) electrical conductivity, and (c) power factor for $Cu_2CoSnS_{4-x}Se_x$ samples with $x = 0$, $x = 1$, and $x = 4$.

Kane band model calculations

To establish the relationship between the carrier concentration and the Seebeck coefficient we employ the solutions of the Boltzmann transport equations within the relaxation time approximation. Considering the Kane band model, the Seebeck coefficient can be found as follows [1,2]:

$$S = -\frac{k_B}{e}\left[\frac{I^1_{r+1,2}(\eta,\beta)}{I^0_{r+1,2}(\eta,\beta)} - \eta\right] \tag{S1}$$

where k_B, e, and r denote the Boltzmann constant, charge of an electron, and scattering parameter, respectively; $\eta = \mu / k_0 T$ and $\beta = k_B T / \varepsilon_g$ are the reduced chemical potential and the parameter determining the degree of band non-parabolicity, respectively. For the case of the parabolic band model, the parameter β was equaled to zero. $I^m_{n,k}(\eta,\beta)$ is two-parametric Fermi integrals:

$$I_{n,k}^{m}(\eta,\beta)=\int_{0}^{\infty}\left(-\frac{df}{dx}\right)\frac{x^{m}(x+\beta x^{2})^{n}\,dx}{(1+2\beta x)^{k}} \tag{S2}$$

The density of electronic states effective mass m^* which was used for the calculations of the Pisarenko relations was estimated using the experimental Seebeck coefficient S and Hall concentration n for each sample:

$$n=\frac{\left(2m^{*}k_{B}T\right)^{3/2}}{3\pi^{2}\hbar^{3}}I_{3/2,0}^{0}\left(\eta,\beta\right) \tag{S3}$$

Elastic properties

The bulk modulus was calculated using the following equation [3]:

$$B=\rho\left(\upsilon_{L}^{2}-\frac{4}{3}\upsilon_{T}^{2}\right) \tag{S4}$$

where ρ is the material density.

The shear modulus was calculated as:

$$G=\upsilon_{T}^{2}\rho \tag{S5}$$

The Young's modulus is calculated as:

$$E=\frac{9BG}{3B+G} \tag{S6}$$

The Poisson's ratio is calculated as:

$$\nu=\frac{E-2G}{2G} \tag{S7}$$

The Debye temperatures were calculated using the following expression [4]:

$$\Theta_{D}=\frac{h}{k_{B}}\left[\frac{3n}{4\pi}\left(\frac{N_{A}\rho}{M}\right)\right]^{1/3}v_{m} \tag{S8}$$

where h is Planck's constant, N_A is Avogadro's number, M is the molecular weight, n is the number of atoms in the molecule, and v_m is the averaged wave velocity integrated over several crystal directions [4]:

$$v_{m}=\left[\frac{1}{3}\left(\frac{2}{v_{t}^{3}}+\frac{1}{v_{l}^{3}}\right)\right]^{-1/3} \tag{S9}$$

where v_l and v_t are the longitudinal and transverse sound velocities, respectively. Grüneisen parameters γ were calculated using the following formula [5]:

$$\gamma=\frac{3}{2}\left(\frac{1+\nu}{2-3\nu}\right) \tag{S10}$$

Thermal transport properties

The phonon mean free paths were calculated by [6]:

$$l_{ph} = \frac{3\kappa_{lat}}{C_V v_m} \tag{S11}$$

Considering the ultrasonic data, the lattice thermal conductivity was also calculated as follows [7]:

$$\kappa_L = K\frac{\overline{M}\Theta_D^3\delta}{\gamma^2 n^{\frac{2}{3}} T} \tag{S12}$$

Here n is the number of atoms in the primitive unit cell, d^3 is the volume per atom, Q_D is the Debye temperature, $\overline{M}$ is the average mass of the atoms in the crystal, and C is a collection of physical constants ($K \approx 3.1\times10^{-6}$ if κ_L is in $Wm^{-1}K^{-1}$, $\overline{M}$ in amu, and d in Angstroms).

According to Cahill's formulation based on the maximum phonon scattering approach, the glass limit for the thermal conductivity κ_{glass} was estimated by [8]:

$$\kappa_{glass} = \frac{1}{2}\left(\frac{\pi}{6}\right)^{1/3} k_B V^{-2/3}\left(2v_t + v_l\right) \tag{S13}$$

where V is the average volume per atom calculated from the refined lattice parameters.

References

[1]. Ravich, Y. I.; Efimova, B. A.; Smirnov, I. A. Semiconducting Lead Chalcogenides; Springer US, 1970. https://doi.org/10.1007/978-1-4684-8607-0.

[2]. Askerov, B. M. Electron Transport Phenomena in Semiconductors; WORLD SCIENTIFIC, 1994. https://doi.org/10.1142/1926.

[3]. Papadakis, E. P.; Papdakis, E. P.; Stickels, C. A.; Innes, R. C. An Ultrasonic Technique for Measuring the Elastic Constants of Small Samples. SAE Trans. 1995, 104, 830–837.

[4]. Anderson, O. L. A Simplified Method for Calculating the Debye Temperature from Elastic Constants. J. Phys. Chem. Solids 1963, 24 (7), 909–917. https://doi.org/10.1016/0022-3697(63)90067-2.

[5]. Sanditov, D. S.; Belomestnykh, V. N. Relation between the Parameters of the Elasticity Theory and Averaged Bulk Modulus of Solids. Tech. Phys. 2011, 56 (11), 1619–1623. https://doi.org/10.1134/S106378421111020X.

[6]. Xie, H.; Hao, S.; Cai, S.; Bailey, T. P.; Uher, C.; Wolverton, C.; Dravid, V. P.; Kanatzidis, M. G. Ultralow Thermal Conductivity in Diamondoid Lattices: High Thermoelectric Performance in Chalcopyrite $Cu_{0.8+y}Ag_{0.2}In_{1-y}Te_2$. Energy Environ. Sci. 2020, 13 (10), 3693–3705. https://doi.org/10.1039/D0EE02323J.

[7]. Morelli, D. T.; Jovovic, V.; Heremans, J. P. Intrinsically Minimal Thermal Conductivity in Cubic I-V-VI_2 Semiconductors. Phys. Rev. Lett. 2008, 101 (3), 035901. https://doi.org/10.1103/PhysRevLett.101.035901.

[8]. Cahill, D. G.; Pohl, R. O. Lattice Vibrations and Heat Transport in Crystals and Glasses. Annu. Rev. Phys. Chem. 1988, 39 (1), 93–121. https://doi.org/10.1146/annurev.pc.39.100188.000521.
